# US Physicians’ Perspective on the Sudden Shift to Telehealth: Survey Study

**DOI:** 10.2196/26336

**Published:** 2021-08-12

**Authors:** Bhavneet Walia, Anshu Shridhar, Pratap Arasu, Gursimar Kaur Singh

**Affiliations:** 1 Department of Public Health Syracuse University Syracuse, NY United States; 2 Division of Cardiology Syracuse Veteran Affairs Medical Center Syracuse, NY United States

**Keywords:** physician survey, US telehealth training, US telehealth care, COVID-19, pandemic, snowball sampling, health care access, health care quality, telehealth, telemedicine, survey, physician, perspective, recommendation, policy, public health, implication, quality, access

## Abstract

**Background:**

Given the sudden shift to telemedicine during the early COVID-19 pandemic, we conducted a survey of practicing physicians’ experience with telehealth during the prepandemic and early pandemic periods. Our survey estimates that most patient visits in the United States during the early COVID-19 pandemic period were conducted via telehealth. Given this magnitude and the potential benefits and challenges of telehealth for the US health care system, in this paper, we obtain, summarize, and analyze telehealth views and experiences of US-based practicing-physicians.

**Objective:**

The aim of this study was to examine the extent of shift toward telehealth training and care provision during the early pandemic from the US-based practicing physicians’ perspective. We also sought to determine the short- and long-term implications of this shift on the quality, access, and mode of US health care delivery.

**Methods:**

We conducted a purposive, snowball-sampled survey of US practicing-physicians. A total of 148 physician completed the survey. Data were collected from July 17, 2020, through September 4, 2020.

**Results:**

Sample training intensity scaled 21-fold during the early pandemic period, and patient-care visits conducted via telehealth increased, on average, from 13.1% directly before the pandemic to 59.7% during the early pandemic period. Surveyed physician respondents reported that telehealth patient visits and face-to-face patient visits are comparable in quality. The difference was not statistically significant based on a nonparametric sign test (*P*=.11). Moreover, physicians feel that telehealth care should continue to play a larger role (44.9% of total visits) in postpandemic health care in the United States. Our survey findings suggest a high market concentration in telehealth software, which is a market structural characteristic that may have implications on the cost and access of telehealth. The results varied markedly by physician employer type.

**Conclusions:**

During the shift toward telehealth, there has been a considerable discovery among physicians regarding US telehealth physicians. Physicians are now better prepared to undertake telehealth care from a training perspective. They are favorable toward a permanently expanded telehealth role, with potential for enhanced health care access, and the realization of enhanced access may depend on market structural characteristics of telehealth software platforms.

## Introduction

### Background

The sudden onset of the COVID-19 pandemic in the United States has provided a test of the US health care delivery infrastructure. As first responders, US health care professionals are navigating a two-pronged challenge not observed in the country since the 1918 H1N1 outbreak—a devastating pandemic that caused at least 50 million deaths globally and was a predecessor to the currently prevalent strains of swine flu. Namely, US health care professionals are treating a largely unknown and deadly virus, while also continuing to practice most other regular functions of medicine during a pandemic. In many cases, technological applications, specifically telehealth, have been drawn on heavily to aid frontline medical professionals in navigating this challenge.

Telehealth represents a potentially cost-effective method to deliver certain types of eligible care, both during and following the pandemic, provided that the underlying technology is able to limit the natural drawbacks of remote care. A primary objective of telehealth is to provide enhanced “access to safe, effective, and appropriate care when and where [patients]. need it, and that providers can [do] more good for more people” [1]. Despite the early recognition of telehealth benefits, US telehealth visits did not scale until many years later. In 2005, there were an estimated 206 telehealth visits in the United States (0.02 per 1000 visits) [2]. By 2017, the estimated number of visits had scaled to 202,374 (6.57 per 1000 visits) [2]. During this growth period, the 2009 American Recovery and Reinvestment Act provisioned $155 billion to US health care toward telehealth-related initiatives such as Health Information for Economic and Clinical Health (HITECH). Despite this rapid growth, data suggest that US telehealth was “still uncommon by 2017” [2], and telemedicine training (eg, in medical school) remained scarce during this period [3-5]. Indeed, physicians reported “considerable interest in, but limited use of, telehealth services” during prepandemic times [6]. Recent infodemiological research suggests that public interest in telehealth was positively correlated to COVID-19 infection rates and that the United States may lack telecommunication infrastructure to meet a growing demand for telehealth [7,8]. More generally, several studies suggest a general rise in telehealth use during recent years [9-13].

### Research Questions and Scope

This study utilizes a large sample US physician survey to characterize changes in the US telehealth use, scale, and training during the COVID-19 pandemic. We also seek to determine the level of physician experience and satisfaction with present telehealth technology platforms and training, as well as diversity of platforms used. Of primary concern, these research questions will help us understand (1) the benefits and challenges of the current telehealth technology, training, and practices; (2) whether physicians believe that recent scaling of US telehealth will sustain in the postpandemic era; (3) whether physicians wish for recent scaling of US telehealth to sustain in the postpandemic period; and (4) how to build more effective telehealth technology infrastructure and practices. Surveyed physicians were asked their *perspectives* regarding telehealth systems used, *helpful and* *challenging* aspects of these systems, and the *overall efficacy of telehealth delivery* compared with *in-person care* for different classes of nonemergency treatments.

We also asked respondents’ *recommendations* for changing the current telehealth systems used vis-à-vis technological features or health care delivery protocol, as well as their vision of the role and scope of telehealth in health care, both during the pandemic and beyond. Beyond this primary research focus, survey responses will provide an estimate as to the diversity of telehealth platforms used across a large sample of physicians. If the telehealth software market features high market concentration among software platform providers, this could cause upward pressure on the price of provisioning telehealth, and potential cost savings from telehealth may not be realized due to market structural factors [14]. The overall effect of telehealth on health care consumer price is indeterminate. For example, telehealth features cost-saving elements that will also be discussed.

### Motivation for Research Design and Research Question Summary

Physician surveys play a vital role toward characterizing health care system inputs and outcomes [15-19], as do systematic reviews of physician survey data [20]. Survey methods have been used broadly to characterize the generally low rate and specialized nature of telehealth adoption (eg, for rural populations) prior to the COVID-19 pandemic [6,21,22]. To a lesser extent, studies have considered the role and scope of telehealth during and after the pandemic [14].

This study seeks to extend the available literature by considering US-based practicing physicians’ views and training and by using characteristics with respect to telehealth expansion during the early COVID-19 pandemic. In doing so, we seek to characterize the implications, benefits, and challenges of this expansion from the perspective of interest, with implications for later-pandemic and postpandemic telehealth provision.

## Methods

### Survey Design

This study presents the first large sample physician survey on telehealth following the onset of the COVID-19 pandemic. We surveyed practicing clinical physicians across the United States regarding their telehealth use and training before and during the pandemic. The survey instrument was constructed by authoring physicians based on their professional experience. The main exclusion criteria for the survey were (1) nonphysician, (2) nonpracticing physician, or (3) physician practicing outside the United States. To obtain a broad, national perspective, we collected surveys from 148 practicing US physicians through snowball sampling and allocated a US $10 e-gift card to each respondent. The snowball sampling methodology was purposive, seeking feedback from US physicians currently using telehealth software. We collected data from July 17, 2020, through September 4, 2020 (ie, for almost 2 months and ending at approximately the half-year point of the US pandemic period).

### Characteristics of Survey Respondents

We divided respondents’ employing organizations into four categories: (1) hospital or larger corporate organization, (2) solo or group practice, (3) government (federal) hospital, and (4) academic hospital. According to the physician respondents, an average of 70% of their patients travelled less than 25 miles for their visit, 18.5% traveled between 25 and 50 miles, and 11.5% traveled more than 50 miles for their visit prior to the pandemic. These values (unweighted means) represent physician *estimates* and are subject to factors such as physician recall and physician knowledge of patient whereabouts. Patients may sometimes choose physicians based on factors such as proximity to work. Furthermore, 28.3% of the patients were aged 51-70 years old, making this the most common age category, and 13.6% of the patients were less than 18 years old, making this the least-represented age category. Patient gender distribution was balanced, with 49.4% female, 48.7% male, and the rest identifying as other. Physicians reported an estimated average payer-mix among patients as 29.2% Medicaid, 31.8% Medicare, 25.5% private health insurance, 10.7% as veterans administrative care, and 2.8% as other.

### Ethical Approval

Data were collected from an anonymous Qualtrics survey that was generated by the authors. The survey was approved by the Syracuse University Institutional Review Board as an ethical research instrument.

## Results

### Training Rates: Overall and by Respondent Characteristic

Survey results on telehealth training vary substantially by physicians’ employer type. Table 1 summarizes the pervasiveness of telehealth training by employer, before and during the pandemic.

Participating physicians report that, on average, their access to telehealth training increased during the early pandemic period as compared to the prepandemic period. The training rate increased in 4 of 5 employer categories (in some instances, it increased dramatically), and it was constant at a high rate in the fifth category. Government hospitals had substantially higher sampled training rates before the pandemic, whereas there was a high degree of convergence in training rates across employers during the pandemic. Sampled physicians report more telehealth training hours during the early pandemic period than during the entire prepandemic period of their respective careers. The average training time increased from 1.33 hours (80 minutes) before the pandemic to 1.67 hours (100 minutes) since the onset of the pandemic.

**Figure 1:**
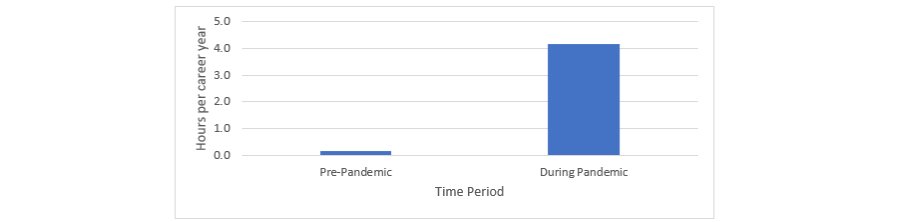
Telehealth training intensity in hours per career year.

Although the general level of the US physician telehealth system training remains fairly modest, these results suggest an abrupt intensification of training during the early pandemic months, which in turn suggests an abrupt shift in the health care system needs and delivery modes with the onset of the pandemic. Figure 1 demonstrates average telehealth training intensities—prorated on a per career year basis—before and during the pandemic period, respectively, where the typical survey respondent had a career length of 8.1 years prior to the pandemic and 0.4 years from beginning of the pandemic to the time of survey (8.5 total career years on average).

Figure 1 shows the extent of shift in telehealth training during the early pandemic period. Sampled physicians reported an average of 0.2 hours of telehealth training per career year before the pandemic and 4.2 hours per career year during the early pandemic period, a 21-fold scaling of training intensity during the latter period.

**Table 1:** Telehealth training coverage by employer type, before and during the COVID-19 pandemic.

Employer type	Telehealth training before the pandemic, n (%)	Additional or first-time telehealth training since the pandemic began, n (%)	Relative increase in training
Hospital or large corporate organization	38 (53.6)	56 (78.6)	Yes
Solo or group practice	10 (27.6)	19 (51.7)	Yes
Government (federal) hospital	11 (80)	11 (80)	Balanced
Academic hospital	12 (43.5)	22 (82.6)	Yes
Other	0 (0)	1 (100)	Yes

### Characteristics of Telehealth Software Used

Regarding telehealth software used, 66.9% (99/148) of the respondents used CloudVisit Telemedicine, 25% (37/148) used Doximity Dialer, and 3.4% (5/148) used Chiron Health. Six other telehealth software were mentioned as the primary software used, where each featured <1% market concentration. This finding suggests a high sample market concentration among telehealth software in the US health care system. The Herfindahl-Hirschman Index (HHI) of market concentration for this sample is approximately 5171. An HHI value of 10,000 reflects pure monopolistic provision, an HHI value between 0 and 1500 indicates competitive provision according to the US Department of Justice, and an HHI value above 2500 indicates a highly concentrated market. These sample results indicate a high level of market concentration in US telehealth software; any limitations of the few leading software platforms will affect physician-patient telehealth interactions at near-market scale. Moreover, market concentration can have profound upward pressure on the price that the software consumer pays and can erode any potential cost savings from telehealth to the health care system and health care consumers. This sample result is consistent with the market concentration characteristics of both software and health care markets.

Questions regarding the general quality of nonemergency telehealth care compared to face-to-face care show that the quality of telehealth was perceived as worse by a plurality of physician respondents (50/148, 33.8%), equal in quality by 31.1% (46/148), slightly better by 14.2% (21/148), much better by 14.2% (21/148), and much worse by 6.7% (10/148) physician respondents. Thus, slightly more sampled physicians reported a quality drop-off rather than a quality gain from telehealth.

### Eligible Visit Types and Intensity of Telehealth Use During the Early COVID-19 Pandemic

Given the challenges and risks of face-to-face visits during the pandemic, physicians are relying more heavily upon telehealth visits. In the prepandemic period, sampled physicians conducted an average of 13.1% of visits via telehealth compared to 59.7% during the pandemic (unweighted means reported). Moreover, Moore et al’s [6] finding that physicians reported “considerable interest in, but limited use of, telehealth services” during prepandemic times is corroborated by our survey data. As several types of visits are not eligible for telehealth delivery, 59.7% of telehealth visits represent an aggressive deployment of telehealth delivery. Physicians have converted approximately 25.9% of telehealth visits into face-to-face visits during the pandemic, down from 32.4% before the pandemic. This finding suggests that, during the pandemic, physicians were using telehealth as a more effective filter in identifying needed face-to-face follow-up visits; patients not needing face-to-face follow-up are more often relegated to telehealth follow-up or no follow-up.

Currently, patient reluctance and internet accessibility represent limiting factors for telehealth provision. On average, physicians reported that 26.6% of the patients are reluctant to participate in telehealth, whereas 29% lack connectivity to conduct a telehealth visit. A description as to the regions served by surveyed physicians may provide context with respect to patient reluctance. The sample represents physicians practicing in 25 US states and 1 US territory, where the sampled physicians serve in all CDC National Center for Health Statistics (NCHS) Urban-Rural Classification categories, including large metro (36/148, 24.3%), large fringe metro (9/148, 6%), medium metro (43/148, 29.1%), small metro (45/148, 30.4%), micropolitan (10/148, 6.8%), and noncore or rural (5/148, 3.4%) areas.

### Telehealth Visit Duration During the Early COVID-19 Pandemic

Of the 148 physicians surveyed, 84 (56.8%) reported allocating the same average time duration to a telehealth visit as to a face-to-face visit; 4 (2.7%), spending substantially less time; 36 (24.3%), slightly less time; 16 (10.8%), slightly more time; and 8 (5.4%), substantially more time. Telehealth does not appear to be substantially distorting the time-of-visit distribution. Overall, more respondents reported allocating less time (40/148, 27%) than more time (24/148, 16.2%) to telehealth visits.

### Characteristics of Physician Views Toward Telehealth

With regard to the postpandemic period, respondents feel that they could deliver approximately 44.9% of patient visits via telehealth. This represents more than three times the reported prepandemic delivery rate for these physicians and only a moderate decrease from the pandemic delivery rate.

Many surveyed physicians (49/148, 33.1%) felt that telehealth delivery decreases the value of their clinical skills, consistent with a capital-labor substitution view of the technology. Loss of patient-physician relationship under telehealth expansion was also a moderately observed response (51/148, 34.5%). It is potentially important to note that patient-physician relationship development may not always be productive in terms of health care. For example, the literature shows that individuals can experience performance decrements as the perceived stakes associated with a task rise [23-25], and that this phenomenon affects surgeons [26]. A surgeon performing a risky surgery may feel more stake-associated pressure if they have formed a relationship with the patient.

According to survey respondents, the five most major challenges faced while providing telehealth during the COVID-19 pandemic are (1) limitations on physician’s ability to deliver certain types of health care by the very nature of telehealth (ie, regardless of level of telehealth development), (2) inadequate telehealth technology, (3) lack of organizational support for telehealth, (4) inadequacies in reimbursement for visit, and (5) prior inadequate physician telehealth training. These results were based on a single question in the survey with categorical response options, as well as a “write-in” reply box. The most common policy recommendations regarding improvements in telehealth delivery were (1) malpractice protection for telehealth, (2) clarity regarding reimbursement policies, (3) training to use technology more efficiently, and (4) policies regarding duration per episode of care. One respondent commented that video-conferencing use, as required by some private insurances, caused problems because many patients were not equipped for videoconferencing.

## Discussion

### Present and Future Use of Telehealth

The results of our physician-respondent survey suggest that the COVID-19 pandemic motivated a substantial shift toward telehealth training and care provision in the United States. Our results further suggest discovery as to the potential and value of telehealth care such that physicians perceive comparable quality of care under telehealth provision compared to a face-to-face visit. Given this discovery, physicians foresee a heavily expanded role of telehealth provision even in the post-pandemic period. However, a moderately high percentage of physician respondents also report both innate and soluble limitations of telehealth technology, as well as a loss of perceived value of their skills under telehealth expansion. Most physicians from different practices and specialties see value in continuing with telehealth provided that a few elements of telehealth provision improve—telehealth technology development, adequate training of physicians and administrative staff, clear reimbursement policies (ie, insurance policies), and clarity on malpractice regulation being the chief elements.

From estimates of patient reluctance and internet accessibility, as reported in the results section, let us conservatively estimate that 30% of the patients have at least one of these limiting characteristics. These limiting factors alone would place a cap on the capacity of telehealth delivery at 70% of visits. Visit eligibility would further decrease this soft cap, such that the survey-estimated pandemic telehealth visit rate (59.7%) represents something close to the present capacity load for telehealth delivery.

Given that physicians feel telehealth care should continue to play a larger role (44.9% of visits) in the postpandemic US health care, we conclude that there was substantial discovery with respect to viability of telehealth during the pandemic period that may not have occurred otherwise. In this respect, the pandemic has been something of a natural experiment for telehealth viability. This also suggests that technological adoption in health care exhibits characteristics of path-dependence or dependence on the occurrence or nonoccurrence of exogenous circumstances rather than being purely a process of optimal decision-making by well-informed firms. Indeed, health care industry policies and practices have been shown to exhibit path dependence [20-22,27-30].

Market concentration is a frequent issue in software markets given that software units can be scaled at essentially zero marginal cost to the seller, and software consumers are often “locked in” after learning a given system. Moreover, we observe high rates of market concentration in health care markets generally, with more than 90% of health care markets characterized as highly concentrated or super-concentrated according to HHI [18]. Telehealth software, which represents a *software* market within the *health care* industry, appears to be no exception. Telehealth software companies sometimes promote further concentration. An article from InTouchHealth, a subsidiary of Teladoc Health, states, “Telehealth would be nearly impossible unless every healthcare provider is using the same system” [1]. This statement represents the monopoly provision of telehealth platforms (eg, via a winner-take-all standards war) as an important condition toward sustained telehealth use by the health care industry. Shachar et al [7] Identified telehealth market regulation as a primary concern with respect to the postpandemic scale and the overall effect of telehealth [7].

### Telehealth and US Health Care Outcomes

The three core objectives of the 2010 US Patient Protection and Affordable Care Act relate to (1) increasing access to health care, chiefly through expanded Medicaid enrollment for the working poor; (2) a higher quality of care through improved medical and market decisions; and (3) reducing cost and patient risk via improved efficiency and higher rates of insured individuals [31]. Our survey results suggest that telehealth provision is largely consistent with these goals. Telehealth has increased access to nonemergency health care during the pandemic and can continue to provide improved access to rural patients and many patients who have difficulties reaching a medical facility in the postpandemic period. Telehealth could reduce the cost of care delivery, as well as the price paid by patients [32] by streamlining some of the logistical hurdles to physicians and patients during face-to-face visits. Furthermore, the survey results suggest that patients often travel long distances for a face-to-face visit. In many cases, this travel time allocation imposes loss of work time and other opportunity costs for the patient that might be minimized through the scaling of telehealth for eligible visits. However, the outcome along this dimension is potentially dependent on market structural and regulatory issues [7]. Survey responses indicate that, on average, the physician-perceived average quality of care remained roughly the same with telehealth expansion. Moreover, telehealth has been popular during the pandemic primarily because it reduces the risk of infection during health care delivery, wherein this risk reduction is an important component of health care quality.

### Takeaways and Future Directions

This study presents a large sample physician survey on telehealth following the onset of the COVID-19 pandemic. The picture that emerges from this physician survey is that the scaling of telehealth can provide the US health care system with increased flexibility, access, and potential health care cost benefits. We acknowledge some study limitations. This was a physician survey and did not capture patients’ views. Further research is therefore needed to determine the benefits and challenges of telehealth expansion from the patients’ perspective. Furthermore, additional research can subcategorize areas of eligible treatment that are more amenable to telehealth expansion in terms of benefits yielded. The survey represents physician responses from 25 US states and 1 US territory. The advantages of the present sample notwithstanding, estimates might have been improved had it been possible to obtain survey responses from physicians in every US state and territory. Moreover, the sample was taken cross-sectionally and does not feature the benefits of a longitudinal survey. More generally, voluntary survey data is subject to recall bias and selection bias, and snowball sampling may lead to sample points that are clustered according to employer or social network. The study also does not address complementary means to improve telehealth infrastructure such as administrative operationalization and clinical care reorganization [33]. Indeed, scaling one’s software capabilities alone will not fully support continued growth in telehealth. Owing to space and scope limitations, such analyses will be considered in future research.

## Abbreviations

CDC: Centers for Disease Control and Prevention
HHI: Herfindahl-Hirschman Index
HITECH: Health Information for Economic and Clinical Health
NCHS: National Center for Health Statistics
